# Design of a 2 DOFs Mini Hollow Joint Actuated with SMA Wires

**DOI:** 10.3390/ma11102014

**Published:** 2018-10-17

**Authors:** Luigi Manfredi, Alfred Cuschieri

**Affiliations:** Institute for Medical Science and Technology (IMSaT), University of Dundee, Dundee DD1 4HN, UK; a.cuschieri@dundee.ac.uk

**Keywords:** 2 DOFs compliant joint, SMA wires in antagonistic configuration, robotics

## Abstract

Shape memory alloys (SMAs) are smart materials used in robotics because of its light weight and high force-to-weight ratio. The low energy efficiency, up to 5%, has limited their use for large actuators. However, they have shown advantages in the design of mini-robots because of the limited volume required for the actuation system. The present study reports the design and construction of a mini compliant joint (MCJ) with a 2 degrees of freedom (DOFs) intersecting axis. The MCJ prototype has a 20 mm external diameter surrounding a cavity of 8 mm, weighs 2 g, is 20 mm high and can perform an angle rotation of 30${}^{\circ }$ in less than 260 ms. It uses SMA NiTi wires in antagonistic configuration and springs to reduce the energy consumption and minimise heat production. The design methods and experimental results of the manufactured prototype are reported and discussed.

## 1. Introduction

The design and construction of a mini-snake-like robot (SLR), particularly for endoscopy, is challenging because of the narrow space available and the total length of the gastrointestinal tract. If the design includes a hollow centre, traditional instruments for biopsy or intervention can be used, which represents an important requirement for the clinical user. Several designs have been reported for SLR devices, most rely on cable-transmission with remote motors controlling each joint, using various control strategies [1,2,3,4]. Clinically, a few SLR have been developed for Minimal Access Surgery (MAS). Choi et al. [5] designed an 8 mm mini-endoscope, 104 mm in length, composed of 7 passive segments, and attached to a spring backbone. The system is equipped with three cables used for actuating the endoscope. The SLR for colonoscope reported by Hu et al. [6], which measures 12 × 600 mm, consists of sections, each actuated remotely by two cables driven by two DC (Direct Current) servomotors.

The design of a SLR using external motors and cables for actuation has certain advantages, including low weight and inertial moment. However, on the downside, each cable has to extend the entire length of the robot for effective control. Additionally, each DOF requires two cables (or a cable/spring combination), which would increase cable friction and the external diameter [7,8,9,10]. For medical applications, the ideal design can be obtained by an actuation system embedded inside the robot [11]. The key issues then concern size and performance of the actuator, which has to meet three requirements: (i) high torque-force-to-weight ratio; (ii) articulated actuation to allow the robot to adapt readily to its surrounding environment; (iii) back-drivability and flexibility allowing passive adaptation of robot shape to that of the surrounding environment without any active control.

A traditional DC or a piezoelectric motor [12,13,14,15,16,17,18], would not be the ideal if the design involves embedding each motor within the robot because of weight and size issues. In contrast, SMA wires, widely used to design joints with multiple DOFs provide a better solution [19]. They can be used in various configurations, including: (i) antagonistic [20,21,22], (ii) with elastic joint [23] and (iii) mixed antagonistic-elastic joint [24,25]. For neurosurgical applications, Ho et al. [26] proposed an SMA actuator based on an active link consisting of two segments: 9 × 13 mm and 10 × 12 mm with a pulley to amplify the SMA movement. In this system, the slow response time (12 to 20 s) required to bend the joint from $0^{\circ }$ to $30^{\circ }$ limits the mechanical bandwidth performance. In the antagonistic configuration, SMA wires can be used with a spring in series for each SMA [27,28]. A recent study [29] proposed to use SMA wires in antagonist configuration serializing two springs, one for each SMA in order to improve SMA reliability by reducing stress. The joint has 2 DOFs with intersecting axes and the SMA wires are in a straight configuration. However, the time to reach a designated configuration is increased because the springs are directly connected to the SMA wires. Additionally, it impacts on the total length of the actuator. Manfredi et al. [30] reported a mini rotary actuator by using SMA wires in antagonistic configuration with on-board position and torque sensors. This study described the use of SMA wires for mini robotics applications requiring a high mechanical bandwidth, up to 10 Hz, with a low power consumption.

The present study concerns a novel patented [31] mechanical design of a hollow joint for the construction of a SLR-endoscope based on use of SMA wires in an antagonistic configuration and intersecting axis. Its design consists of a primary and secondary transmission decoupled by a symmetrical torsional spring to improve the performance in terms of energy efficiency and heat reduction. The frame is a simple and compact homo-kinetic hollow joint with a light but stiff structure. The output torque varies with the diameter of the SMA wires used, although to ensure a high mechanical bandwidth this should not exceed 100 μm. The joint was constructed by using a 3D printer and open-loop step response experiments are reported.

The described design offers three advantages compared with the previous reported studies of rotary actuators by using SMA in antagonistic configuration: (i) internal cavity for cabling or instruments channels, (ii) compact design by using pulleys to reduce the overall length and provision of 2 DOFs; (iii) inclusion of a torsional spring to confer a compliant behaviour with a low power consumption.

## 2. The Mini Compliant Joint Design

**Figure 1:**
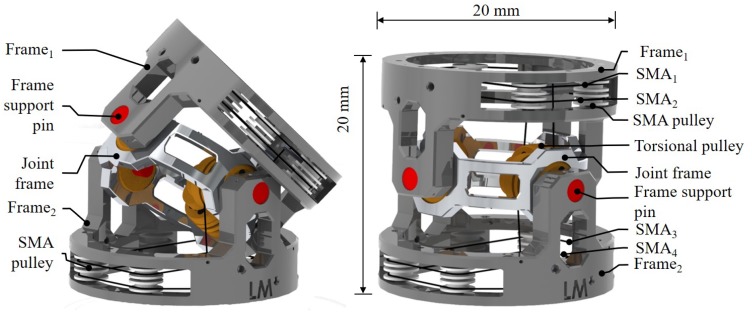
Frame of the 2 DOFs MCJ. CAD design, 20 × 20 mm and an internal hollow area of 8 mm diameter. It has two intersecting axes, roll ($\beta_{1}$) and pitch ($\beta_{2}$) (counter-clockwise rotation around the *X* axis and counter-clockwise rotation around the *Y* axis), each joint is actuated by means of two SMAs wires in antagonist configuration with a torsional spring to decouple the joint in order to achieve a compliant mechanism, reduce the heat and increase the efficiency of the system. The 1st DOF is controlled by activating SMA${}_{3}$ (clockwise) and SMA${}_{4}$ (counter-clockwise), while the 2nd DOF by SMA${}_{1}$ (clockwise) and SMA${}_{2}$ (counter-clockwise).

The joint design is symmetric with 2 DOFs using 2 SMA wires in an antagonistic configuration with a torsional spring for each DOF; thus, comprised of 4 wires and 2 springs in total as it is shown in Figure 1. The torsional spring decouples the SMA wires from external forces, thereby providing a flexible and passive compliant behaviour, with reduced energy consumption, diminished heat generation and a safe stable mechanism [32]. Additionally, the spring reduces the impact of any external torque on the system increasing the SMA life [29]. With the SMA wire in antagonistic configuration, the operative range of each joint is related to the length of the wire, which can be $\epsilon_{S}$ = 5–7% of its total length [33]. To reduce the length of the joint, the SMA wire is coiled inside the frame using pulleys (accommodating the wire around the shape of the frame) in order to decrease the friction, hence increase overall efficiency.

**Figure 2:**
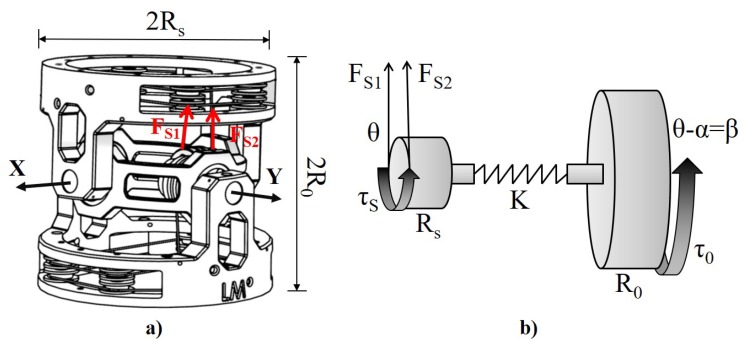
(**a**) Joint with 2 DOFs, where $\beta_{1}$ and $\beta_{2}$ are the angles around the 2 intersecting axes X and Y, with a desired range of $30^{\circ }$; $2R_{S}$ is the diameter and $2R_{0}$ the length of the joint. $F_{S1}$ is the force produced by SMA${}_{1}$ with a clockwise rotation, and SMA${}_{2}$ produces a force $F_{S2}$ and a counter-clockwise rotation. (**b**) Joint working principle, where a torque $\tau_{SM}$ is the result of two forces $F_{S1}$ and $F_{S2}$. The output torque, $\tau_{O}$ is decoupled from $\tau_{SM}$ by means of a torsional spring to reduce the energy consumption and stress on the SMA wires.

The actuator is composed of three main parts as shown in Figure 1: an upper frame (Frame 1), a lower frame (Frame 2) and a central frame (Joint frame), which functions as a link between the upper and the lower frames. As the configuration is symmetric, only the upper frame is described. Each frame has a pulley actuated by means of the 2 SMA wires in antagonist configuration. The pulley is linked to the central frame by a torsional spring resulting in a passive compliant joint as shown in Figure 2b and described in detail in Section 2.1. The joint design was optimised by Finite Element Analysis (FEA) to define the thickness and shape to reduce the weight of the joint, before its initial production by a 3D printer. In the final version, the external diameter and length are equal and measure 20 mm. The weight of the frame without the electronic system is 2 g.

Both SMA wires can be independently controlled to achieve the desired angle and to change the stiffness of the system by regulating the temperature of each wire. The stiffness can be adjusted by pulling simultaneously the two SMA wires in an antagonistic configuration, adjusting the force applied by each wire, although this requires precise control of the current provided to each SMA wire. The choice of the spring properties defines the stiffness of the system of the compliant joint.

The torsional spring located between the two pulleys decouples the primary from the secondary pulley. The torsional pulley can provide a maximal torque of 3 Nmm.

### 2.1. Working Principle

The joint has 2 DOFs with 2 intersecting axes, X and Y as shown in Figure 2a. A pair of SMA wires in antagonistic configuration is required to control each axis; 4 SMA wires in total to assemble the joint. The working principle of each axes is showed in Figure 2b and Equation (1) describes the statics when $\tau_{S}=-\tau_{O}$:

(1) $$\tau_{S}=\left(F_{S2}-F_{S1}\right)\times R_{S}=K\times \alpha =-\tau_{O}$$

(2) $$K=\frac{-\tau_{O}}{\alpha_{MAX}}=\frac{\tau_{S}}{\alpha_{MAX}}$$

where $F_{S1}$ and $F_{S2}$ are the forces produced by each SMA wire, $\tau_{O}$ is the output torque, $\tau_{S}$ is the resultant torque produced by the SMA wires antagonist configuration, *K* the torsional spring elastic constant (considering the Hooke’s law for a linear spring), $R_{S}$ and β are the radius (6 mm) and the angle of the torsional pulley, $R_{0}$ is half of the joint length (10 mm), α the angle of the torsional spring, $\alpha_{MAX}$ the maximal compliance angle. Equation (2) defines K of the torsional spring to achieve the maximal compliance angle $\alpha_{MAX}$.

When the power supply is off, an SMA wire provides a residual force of about $F_{MAX}/3$ [24,34], where $F_{MAX}$ is the maximal contracting force, which equates to a residual torque of $\tau_{S}/3$. This residual force has been experimentally investigated in Section 3.3.

(3) $$F_{S}=F_{MAX}-\frac{1}{3}F_{MAX}=\frac{2}{3}F_{MAX}$$

This allows the joint to have a residual compliance of $\alpha_{MAX}/3$ with no need for external power and a consequent reduction of energy consumption. Each joint can provide an output torque $\tau_{O}$ as described by the equation:

(4) $$\left(\frac{2}{3}F_{MAX}\right)R_{S}=\tau_{MAX}$$

which is reduced by circa 1/3 due to a residual strain of the opposite antagonistic SMA wire when it is not powered. The residual torque can be adjusted by controlling the temperature of the SMA to increase joint stiffness.

The performance of the system is determined by a balanced interaction of three parameters: diameter of the SMA wire $d_{SMA}$, diameter of the pulley $2R_{S}$, and the joint length $2R_{0}$, which in turn determine the output torque $\tau_{O}$, the output force $F_{0}$, the angular motion range $\beta_{MAX}$, the arc range of motion $L_{MAX}$, the mechanical bandwidth $f_{MAX}$ of the system, the angular velocity $\omega_{MAX}$ and the radial velocity $v_{MAX}$.

Increase in $d_{SMA}$ decreases $\tau_{O}$, $\omega_{MAX}$ and the radial velocity $v_{MAX}$. Additionally $f_{MAX}$ will be reduced because the thicker wire increases the response time. Both the $\beta_{MAX}$ and the $L_{MAX}$ will not be altered.

Increase of the pulley diameter $2R_{S}$, reduces the output parameters $f_{MAX}$, $\beta_{MAX}$, $L_{MAX}$, $\omega_{MAX}$; $v_{MAX}$ will be reduced but the $\tau_{O}$ will increase because of the higher cantilever configuration.

$R_{0}$ increases directly both $L_{MAX}$ and $v_{MAX}$ without any effect on the other parameters. Table 1 shows the correlation between the system design parameters and the system output performances.

### 2.2. Design of SMA Wires

The length of the wire is crucial to the overall design since this impacts on the range of motion of the joint $\pm \beta_{MAX}$. If a reasonable range of joint motion needed is of $30^{\circ }$, the extension of the SMA wire is described by the equation:

(5) $$\begin{array}{cc} \Delta L & =2\beta_{MAX}R_{S} \end{array}$$

where ΔL is the SMA elongation, $\pm \beta_{MAX}$ is the joint range, and $R_{S}$ is the radius of the SMA pulley. Assuming $\beta_{MAX}=15^{\circ }$=15×π/180 radians, and $R_{S}=2$ mm, ΔL is described by:

(6) $$\begin{array}{cc} \Delta L & =2\times 15\times \pi /180\times 2\\ & =1.03\;\mathrm{mm} \end{array}$$

and elongation described by Equation (6):

(7) ΔL=ϵL

(8) $$\begin{array}{cc} L & =\frac{\Delta L}{\epsilon }\\ & =\frac{1.05}{0.03}\\ & =35\;\mathrm{mm} \end{array}$$

Hence 35 mm is the required minimum SMA wire length, with ϵ=3% being considered as a precautionary limit. The reason for this consideration is also related to the mechanical stability of SMA wires. This mechanical behaviour has not been addressed in this study although this is related to the strain that can be recovered when the SMA is activated therefore the range of motion of the actuator. Several studies on SMA fatigue have shown limited degradation with a maximal strain of 4% after 5 × 10${}^{5}$ cycles [35,36].

**Figure 3:**
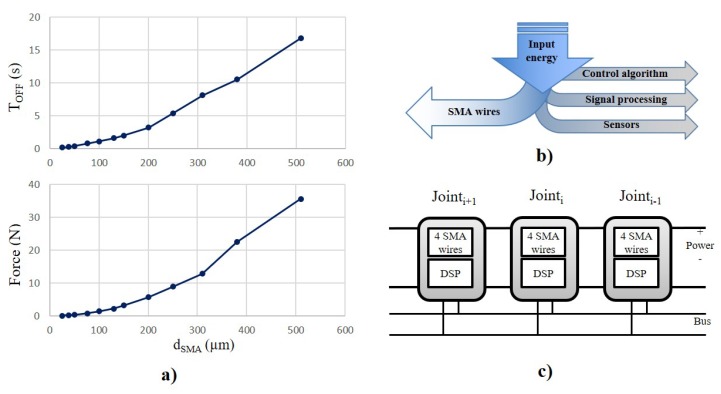
(**a**) SMA wire Flexinol${}^{®\;}$ produced by Dynalloy${}^{®\;}$ Inc. performance in terms of SMA wire diameter vs. Force (top graph) and SMA wire diameter vs. $T_{OFF}$ (bottom graph). To achieve a mechanical bandwidth above 1 Hz, the SMA wire diameter should not exceed d<100 μm. (**b**) In the worst scenario, up to 95% of the power supplies the SMA wires. Control algorithm, signal processing and sensors use only a minimal amount of the overall energy available to the system. However, this increases when the SMA wires are not activated and the mechanical system is moving passively by the compliance behaviour provided by the torsional springs. The advantage of this passive mechanical solution is that when the SMA wires are not active, the energy consumption sink from the actuation system is zero. (**c**) Proposed distributed control hardware using a parallel bus (e.g., I2C) and power, to achieve a modular system and to simplify the cabling of the robot. Each module is able to control one joint by using a closed-loop position control. This hardware is connected to an external PC and data exchanged by means of an I2C communication bus.

To achieve a compromise between a low power consumption but a good mechanical bandwidth, SMA wires used in this design have a low temperature profile of $70{\;}^{\circ }$C (Flexinol${}^{®\;}$ produced by Dynalloy${}^{®\;}$ Inc., Irvine, CA, USA) with a mechanical performance, heating and cooling time, reported in Table 2. SMA wires with higher temperature profile have a higher mechanical bandwidth although they require a higher activation current. This phenomenon is related to the higher difference in temperature between the SMA wires and the environment, which increases the heat dissipation and therefore it reduces the deactivation time, $T_{OFF}$ in Figure 3a. The robust nature of the frame, permits use of different wire gauges. To achieve a high bandwidth, the SMA wires selected have a diameter of 50, 76, 100 μm because the switch on-off time varies according to diameter of wire, i.e., 1.0–0.3 to 1.0–0.5, and 1.0–0.8 s. Thicker SMA wires can be employed but would incur increased energy consumption and reduction of mechanical bandwidth [30].

An SMA wire of length *L* and diameter *d*, will have a volume proportional to L×d and a surface to $d^{2}$, therefore unit surface per volume is proportional to $d^{-1}$. A narrow wire is desirable because the heat generated is proportional to the surface area of the wire.

**Figure 4:**
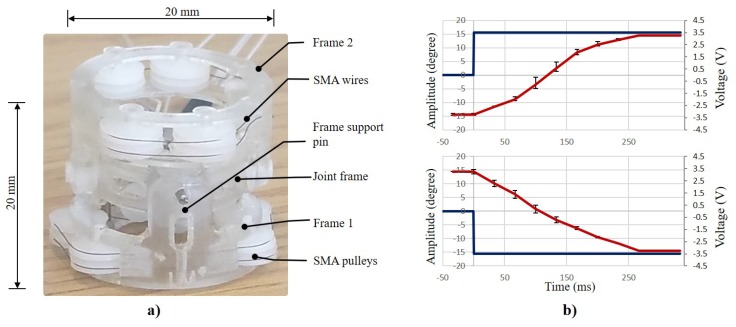
(**a**) Prototype of the miniaturised compliant 2 DOFs 3D printed joint composed of frame 1 and 2, a joint frame, pulleys and four SMA wires. (**b**) MCJ time vs. amplitude when a step voltage profile from 0 to 3.5 V is applied to each SMA wire, performing a position control moving the joint clockwise (top graph), SMA${}_{3}$-ON, SMA${}_{4}$-OFF, and counter-clockwise (bottom graph), SMA${}_{3}$-OFF, SMA${}_{4}$-ON, showing mean and standard deviation values of the angle position.

Figure 4a reports two graphs taken from the Dynalloy${}^{®\;}$ SMA wires datasheet, *d* vs. $T_{OFF}$ time, and *d* vs. $F_{OUT}$. To achieve a mechanical bandwidth of 1 Hz, the SMA wire requires a diameter of d<100 μm (Figure 4a-top graph; it can produce a force of $F_{OUT}<1.5$ N (Figure 4a-bottom graph), thus achieving a good mechanical bandwidth.

The mechanical property of the spring was selected to have a compliance range up to $\pm 10^{\circ }$ when the maximal torque $\tau_{MAX}$ is applied to the system.

The overall size of the joint is reduced by using pulleys although they can increase the fatigue stress of the SMA wires, with a consequent reduction of the life time of the joint. The bending stress produced by a pulley is described by the following equation:

(9) $$\epsilon_{max}=\frac{d/2}{R_{S}+d/2}$$

where $\epsilon_{max}$ represents the maximal normal strain.

### 2.3. Powering and Heating

One issue of SMA is its low energy efficiency, up to 5–7%, which has limited its use in actuator systems. The low efficiency induces heat generation which in turn alters the resistance of the SMA, described by:

(10) $$\rho =\rho_{0}L$$

where $\rho_{0}$ is the resistance per cm, *L* is the wire length, and ρ is the resistance of the whole wire. In the worst case scenario at full power up to 95% of this energy is lost as heat in the SMA wires.

Table 3 illustrates the resistance values of the wires used in the design. The power consumption of the actuator is not a constant value, because it depends on the heat dissipation, being higher initially and then reducing slightly [37]. In the present experiments, the power reached 212–525–700 mW (Table 4). Future R&D will be directed to optimization of the control to increase the efficiency of the SMA. In a robot design the compliant behaviour of the component actuators reduces the need for keeping the SMA wire active due to the passive mechanical behaviour of the springs, which reduces the energy required for locomotion (Figure 3). In addition, previous studies reported by the authors have shown a low power consumption by a rotary actuator whilst maintaining a desired angular position [30,38,39].

## 3. Results

### 3.1. Frame Prototype

The design intentionally achieved a light joint with a high output torque. Hence particular attention was paid to produce a light but robust frame. The first prototypes were constructed by 3D printing (Stratasys Ltd.®, Eden Prairie, MN, USA), using Polyjet RGD525 polymer (Table 5). This approach enabled construction and testing of several prototypes, with the frame weight being gradually reduced by removing material in the unstressed sections as indicated by the FEA.

**Figure 5:**
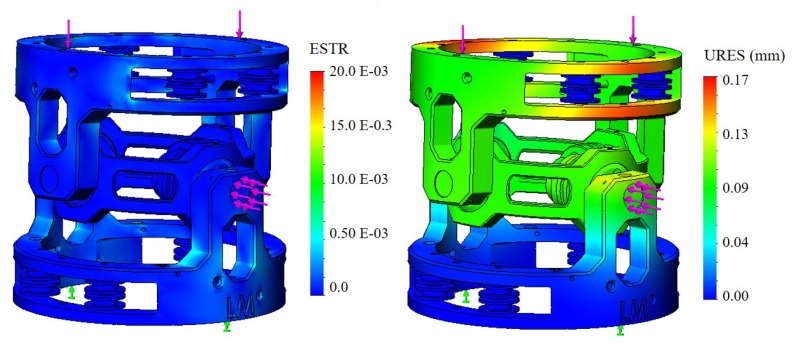
FEA: two forces of 10 N each, are applied on the top and lateral side of the joint, as shown in the figure. The left picture depicts the strain stress, and the right, the displacement.

The analysis was performed using SolidWorks simulation software (2016). External forces were applied to 2 sections of the frame. One section is the SMA wires with connection to the main pulley because of the stress produced by the wires when activated. The other is top section of the frame to which one or more MCJs will be connected. In the analysis, the bottom section of the MCJ is defined as fixed. Figure 5 shows the FEA of the MCJ applying 2 forces of 10 N each, superior to and lateral to the frame (violet force in the figure) and the fixed section (green force). The simulation reports the ESTR and URES values, ESTR representing the equivalent strain and the URES, the U value of the resultant of the displacement in millimetres.

The frame weight of the final prototype is 2 g. In order to reduce the joint friction, four miniature pins (thickness of 1.5 mm) were constructed using Delrin®. Pulleys around the frame served to reduce both the overall length of the actuator and the friction SMA wires fixed to each frame. This allowed coiling of the wires around the frame. The size of the pulley influences the level of friction and hence the efficiency of the joint. Increase in diameter would reduce friction, although this would be at the expense of internal joint space. The joint frame is connected to the upper and lower frame by means of 4 frame support metal pins.

### 3.2. Electronic and Control

As with the mechanical design, the electronic control needs to be located in each independent joint, embedding all the required hardware to control the 2 DOFs as shown in Figure 3c. For this reason, the electronic system must consist of a distributed nature, using a parallel bus to exchange control data.

A preliminary prototype of the control hardware has been implemented with an off-the-shelf electronic board to investigate the performance of the MCJ. It consists of a 16 bits DSP (Digital Signal Processor) Microchip, where the low-level control is implemented in C code. It uses an open-loop control that changes the duty cycle of the PWM (Pulse Width Modulation) of the output voltage to each SMA wire. The firmware receives commands from an external PC (Personal Computer) through an I2C bus protocol. Four wires are needed for the actuator: two providing power, and two for a serial communication bus. Four MOSFET (Metal-Oxide-Semiconductor Field-Effect Transistor) and current sensors are used to regulate the power for each wire. In the snake configuration made with several Miniature Compliant Joints (MCJs), a mid-level control will be implemented in a main control board at the distal end of the robot. In view of the requirement for fast advanced computation, high-level control e.g., as inverse kinematics, navigation and image processing, will be provided by an external PC.

### 3.3. SMA Force and Current Profile

**Figure 6:**
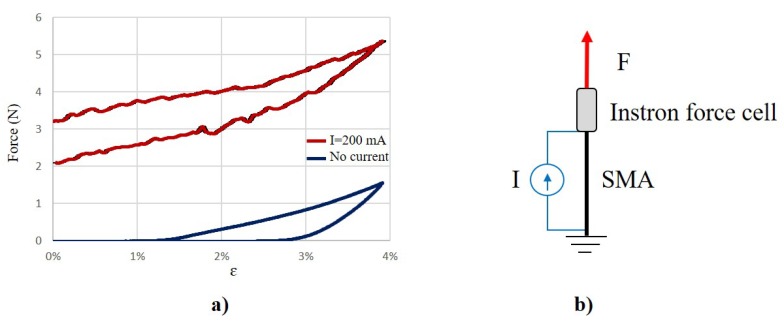
(**a**) Stress vs. strain profile of an SMA wire with a diameter of 100 μm when the wire is not activated (blue line) and when it is activated with 200 mA of current. (**b**) Experimental set up for testing of the SMA wire by connecting one end to both an external power supply and the Instron. The other end is grounded.

Experiments to define the residual strain and force have been performed. An SMA wire with a diameter of 100 μm at room temperature has been tested by using an Instron${}^{®\;}$ 5564 dual columns (High Wycombe, Buckinghamshire, UK). One end of the SMA wires was attached to the force cell (50 N) and to an external power supply. The other end was connected to a grounded base. The rest position was considered as the length of the SMA wire after heated with 150 mA for 5 s and cooled down for 1 min. The Instron was programmed to provide a ramp position profile with a speed of 0.05 mm/s from the original position up to a strain of 4%. The experiments were repeated 5 times each and the mean value plotted into the graph as shown in Figure 6, when no current (blue line) and 200 mA of current (red line) was applied. It is shown that the maximal force produced by the wire when activated (5.35 N) is more than 3 times higher than the residual maximal force produced by the SMA wire when it is not activated (1.56 N). This residual force is used to design the actuator to keep a commanded position reducing the needed power and performing a compliant behaviour, as described in Section 2.1.

Figure 6a shows the characteristic hysteresis profile of SMA wires. The blue line shows wire when not activated (in the martensite). The external force produced by the Instron stretches the wire with a limited recovery of the original shape on unloading. The activated wire shown by the red line is associated with an increase in the temperature and transition from martensite to austenite. The increase of the external force stretches the SMA wire. When the Instron force is reduced, the phase wire changes from the austenite to the martensite phase. This transition is responsible for the hysteresis in the force vs. strain profile.

### 3.4. Angle Position Profile

Figure 4a shows final prototype constructed by 3D printing and tested using three different SMA wires to assess the mechanical performance of the actuator. Only 1 DOF was activated and tested since the design has a symmetry and the mechanical principle is the same. The prototype compliant actuator consists of three parts: upper frame (Frame 1) and lower frame (Frame 2) both with a diameter of 20 mm, and a length of 11 mm, and a linker (15.2 mm diameter), which connects both frames by four 1.5 mm pins and two torsional springs, as shown in Figure 4a. All the experiments were performed at room temperature and by using low temperature (LT) standard Flexinol${}^{®\;}$ actuator SMA wires produced by Dynalloy${}^{®\;}$ Inc. (Irvine, CA, USA). The power provided for each 40 mm wire is shown in Table 4. The mean response time of the system is below 260 ms, which is also the time taken to achieve an angle of $30^{\circ }$. During these preliminary experiments, no heating problems were encountered.

Figure 4b shows a rotation angle profile exhibiting a good mechanical bandwidth and range of motion. The experiments were performed three times for each angle profile, and the graphs report mean values and standard deviation. The MCJ is actuated by supplying each 100 μm SMA wire with a 3.5 V step voltage profile and a maximal current of 200 mA. A power ground is connected to the junction of the 2 SMA wires next to the pulley inside the joint frame. Both angle profiles (counter-clockwise and clockwise), show a similar performance. The asymmetric profile is related to a small difference in the length of each activated SMA wire, which translates into different resistances and therefore different current supplied. This limitation can be avoided by the use of a closed-loop control with and on-board position sensor [30].

## 4. Discussion and Conclusions

A 2 DOFs joint (20 × 20 mm), with an internal diameter of 8 mm, and weighing 2 g is described. It is actuated by 4 SMA wires in antagonistic configuration with 2 torsional springs and a range of $30^{\circ }$. The preliminary experiments have shown that the actuator design provide a good performance, including mechanical bandwidth, power supply, and light weight.

The experiments with different SMA wires confirmed generation of a wide range of forces needed to cope with different frame robustness. SMA has a low energy efficiency that can vary from 5% to 7%, therefore there is a requirement for an efficient system of heat dissipation. Several considerations were necessary to achieve the final design to minimise overheating. The MCj’s frame can be made of metal and can be used to dissipate the heat produced considering that the mass of the SMA wires is negligible compared to that of the joint. An advantage of this design in reducing the heat production is the use of a passive compliant system by means of a torsional pulley. This solution allows the SMA wires not always to be active, reducing overheating, without compromising its compliant behaviour. Even so, the overall temperature needs to be monitored. The low-level control can monitor the temperature and reduce the performance in the event of overheating. A flexible skin is also needed to protect the mechanism of the joint and to avoid any electrical leakage with an external material. Also, the pulleys where the SMA wire is in contact require to be constructed from non-electrically conductive material.

When more joints are connected, to form a tethered snake-like robot, the high-level locomotion control can reduce the overall heat using different strategies, by considering a residual torque of each joint when the power is off. Another strategy would be avoiding simultaneous activation of all the joints; instead it will ensure sequential activation or activation only of the advancing joints at the tip of endoscope. In essence, the environment will not be affected by overheat for the following reasons: (i) the SMA wires are not exposed and are not touching the external environment; (ii) the negligible mass of the SMA wires compared to the frame allows to dissipate the excessive temperature by using metal; (iii) the passive compliant mechanism reduces the need for keeping the SMA wires always active; (iv) we have proved in a previous study that the required energy to keep a desired angular position with SMA in antagonistic configuration is low [30].

The frame was confirmed to be mechanically stable without any evidence of mechanical backlash. The system for coiling the SMA inside the frame requires improvement, possibly by using a different material for the pulleys together with improved manufacturing to reduce the friction and to ensure smooth rotational movements and durability, e.g., by using a miniaturised ball bearing inside each pulley. Other improvements are needed in relation to (i) manufacturing process and (ii) the electronics system including actuation control strategy.

The manufacturing process can be improved by using different materials (e.g., aluminium, titanium), which are lighter and stronger, to reduce the weight and increase heat dissipation. The current experiments have confirmed that the SMA wires can be coiled inside the frame by means of small pulleys to reduce the friction and the overall dimensions of the joint. In this context, the size and friction of the pulley is crucial to the smooth performance of the actuator.

Different control strategies can be implemented. This includes the use of a sensor-less position and torque joint control [30,37]. The efficiency and the system dynamics can also be improved by adjusting the current provided to each wire. This will however require cooperative control of the antagonist SMA wires to adjust the stiffness of the actuator. The efficiency and the system dynamics will benefit from improved control of the current provided to the wire. The energy consumption of each joint requires a tether to supply the power needed, which can also be used to stream video images captured by an on-board camera.

## 5. Patents

Aspects of this technology are protected by GB Patent Application GB201407490D0.

## Figures and Tables

**Table 1:** Correlation of the system design parameters.

	$d_{\mathrm{SMA}}$	$R_{S}$	$R_{0}$
$\tau_{O}$	↑	↑	=
$f_{MAX}$	↓	↓	=
$\beta_{MAX}$	=	↓	=
$L_{MAX}$	=	↓	↑
$\omega_{MAX}$	↓	↓	=
$v_{MAX}$	↓	↓	↑

**Table 2:** SMA wires performances in terms of heating, cooling time, and output force, used in the proposed joint design in antagonist configuration.

Size $d_{\mathrm{SMA}}$		Total Length	Time ON/OFF	Force
(mil)	(μm)	(mm)	(s)	(mN)
2.00	50.8	40	1–0.3	350
3.00	76.2	40	1–0.5	800
4.00	101.6	40	1–0.8	1500

**Table 3:** SMA resistance for a 40 mm wire long, which is the worst scenario when both SMA wires are activated.

$d_{\mathrm{SMA}}$		Resistance	Length	Resistance
(mil)	(μm)	(ohm/cm)	(mm)	(ohm)
2.00	50	4.72	40	18.9
3.00	76	1.97	40	7.87
4.00	100	1.18	40	4.72

**Table 4:** SMA powering experiment text.

Size		Current	Voltage	Power
(milliinch)	(μm)	(mA)	(V)	(mW)
2.00	50	85	2.5	212
3.00	76	150	3.0	525
4.00	100	200	3.5	700

**Table 5:** Mechanical properties of the Polyjet RGD525 used for the manufacturing of the frame.

	Units	Metric	Units	Imperial
Tensile Strength	MPa	70–80	psi	10,000–11,500
Elongation at Break	%	10–15	%	10–15
Modulus of Elasticity	MPa	3200–3500	psi	465,000–510,000
Flexural Strength	MPa	110–130	psi	16,000–19,000
Flexural Moduls	MPa	3100–3500	psi	4,500,000–510,000
Polymerized Density	g/cm${}^{3}$	1.17–1.18		

## Abbreviations

The following abbreviations are used in this manuscript:

SMA	Shape Memory Alloy
MCA	Mini Compliant Joint
DOF	Degree of Freedom
MAS	Minimal Access Surgery
SLR	Snake Like Robot
FEA	Finite Element Analysis
